# Supplementary material for: CRISPR/Cas9‐mediated resistance to cauliflower mosaic virus
Source: Plant Direct. 2018 Mar 7;2(3):e00047. doi: 10.1002/pld3.47 (PMC6508564; doi:10.1002/pld3.47)
Supplement: Supplementary file 1 [file PLD3-2-e00047-s001.pdf]

taccccaattacggagtaggaaatgaagaattaggatcctctggaaaccctaaagctttaacctggcccttcaaagctccagcaggatggccgaatcaa
ttttag**acaggaccattaaccggttctgg**tataatctgggagaagattgtctctcagaaagtcaattcgatcttatgatcagattaatggaa
gagtcccttgacggggaccaaattattgatctaacctctctacctag**tgataatttgcaggtcgaacagg**ttatgacaactaccgaagac
tcgatctcggaagaatcagaattccttctagcaataggagaaacat**ctgaagacgaaagcgattcagg**agaagaacctgaattcgag
caagttcgaatggaccgaacaggaggaacgg**agattcccaaagaagaagatgg**tgaaggaccatcaagatacaatgagagaaa
gagaaagaccccggaggaccggtacttt***ccaactcaaccaaagaccattc***cag**gacaaaagcagacgtccatagg**aatgctca
acatcgactgccaagccaatcgaagaactttaatcgatgactgggcagcagaaatcggattgatagtcaagaccaatagagaagacta
tcttgatccagaaacaattctactcttgatggaacacaaaacatcaggaatagccaaggagttaatccgaaatacaagatggaaccgca
ctaccggcgacatcatagaacaggtgatcgatgcgatgtacaccatgttcttaggactaaactactccgacaacaaggttgctgagaag
attgaagagcaagagaaggccaagatcagaatgaccaagctccagctctgcgatatctgctaccttgaagaatttacatgtgattatgaa
aagaacatgtacaagacagaactggcggatttcccaggatatatcaaccagtacctgtcaaaaatccccatcattggagaaaaagcgtt
aacacgctttagacatgaagccaatggaaccagcatctacagcttaggtttcgcggcaaagatagtaaaagaagaactatctaaaatct
gcgacttaaccaagaagcagaagaagttgaagaaattcaacaagaagtgctgtagcatcggagaagcttcagtagaatatggatgca
agaagacatccaagaagaagtatcataaaagatacaagaaaaaatataaggtctataaaccttataagaagaagaagaaattccgtt
ccggaaaatacttcaagcccaaagagaagaagggctcaaagcaaaaatattgcccaaaaggcaagaaagattgcagatgttggatc
tgcaatatcgaaggccattacgccaacgaatgtcctaatcgacaaagctcggagaaggctcatatccttcaacaagcagaaaatttggg
tctacagcccattgaagaaccctatgaaggagttcaagaagtattcatcttagaatacaaagaagaggaagaagaaacctctacagaa
gaaagtgatggatcatctacttctgaagactcagactcagactgagagcaggtgatgaacgtcaccaatcccaattcaatctacatcaagggaag
actctacttcaaaggatacaagaagatagagcttcattgtttcgtagac

Figure S1 The sgRNA sequences in relation to CaMV coat protein open reading frame (from strain w260, sequence ID: JF809616.1, length: 8041). Targeting sequences of six sgRNAs are in bold and PAM sites are underlined. Note the italicized sequence is target on the complementary strand. The sequences in purple are the ORF of *CP*, the sequences immediately upstream and downstream of *CP* are shown in black with PCR primers sequences in green.

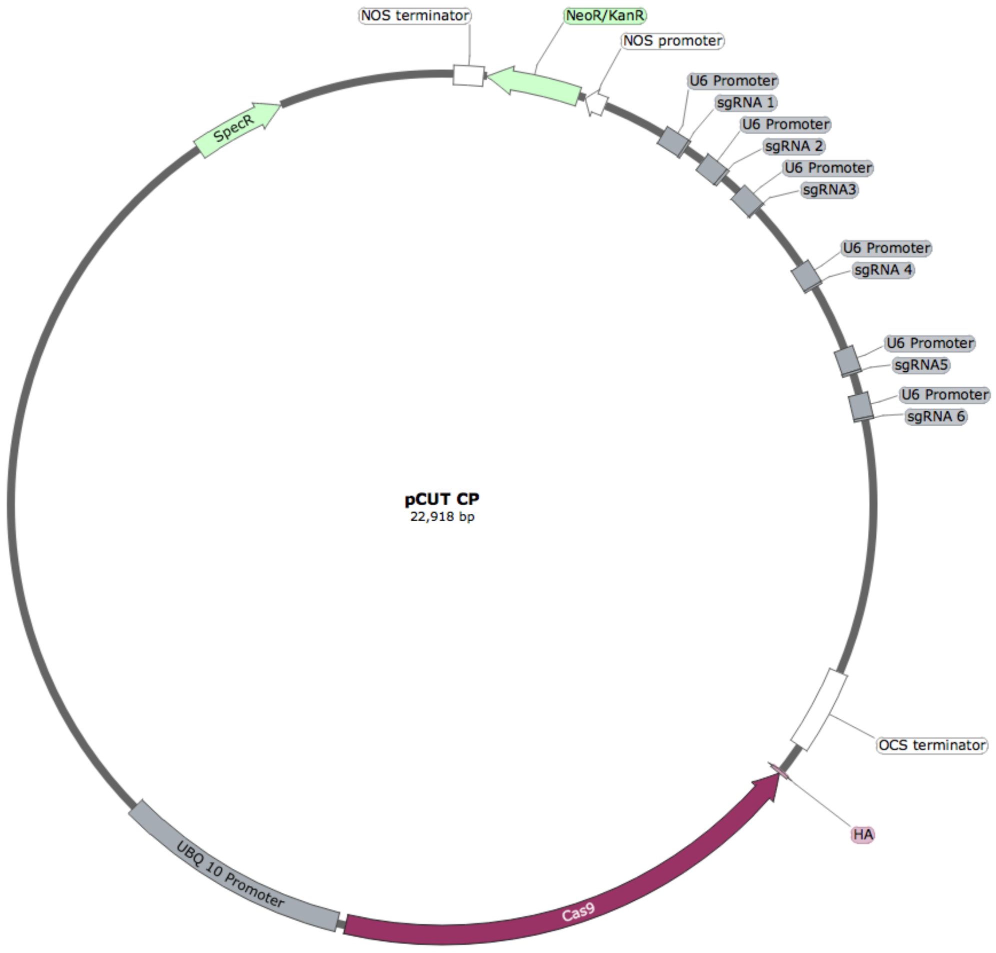

Figure S2. The map of *pCUT CP*. The expression of Cas9 is under the control of the *UBQ 10* promoter and 6 sgRNAs is under the control of the *U6* promoter.

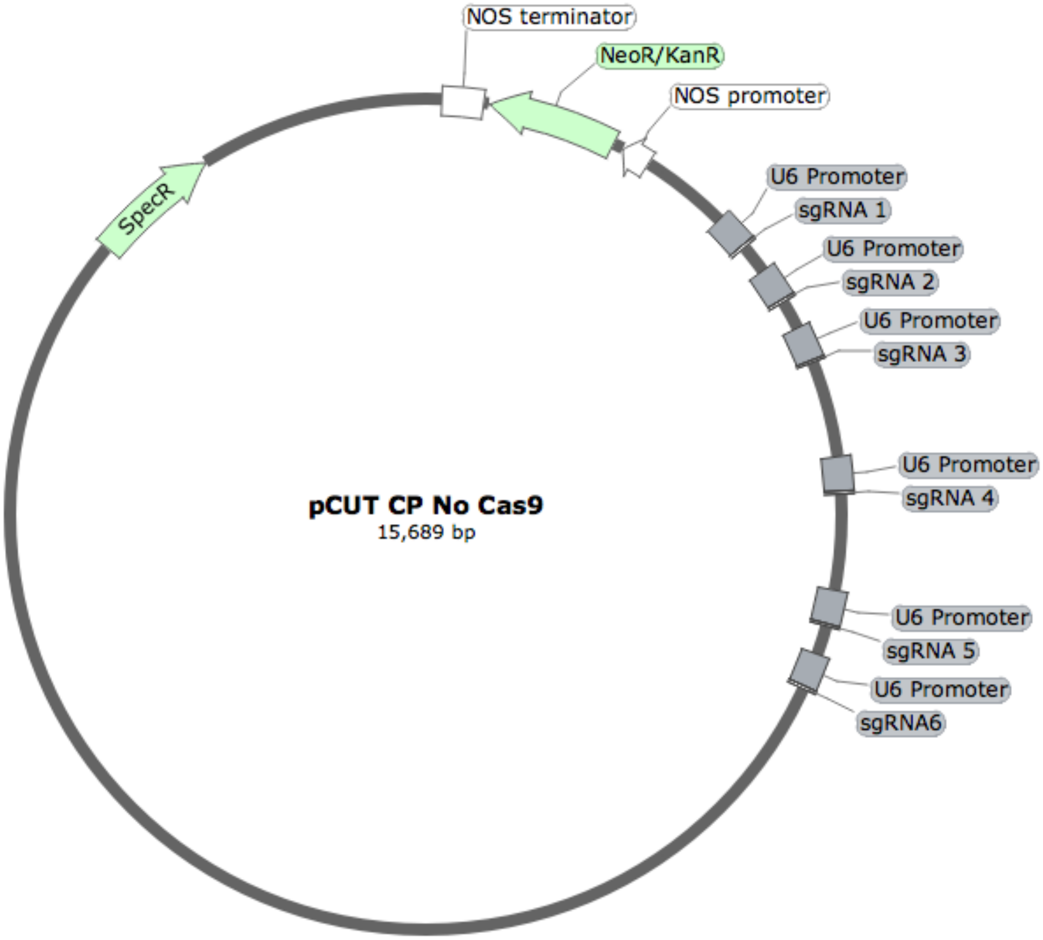

Figure S3. The map of *pCUT CP No Cas9*. The expression of 6 sgRNAs is under the control of the *U6* promoter.

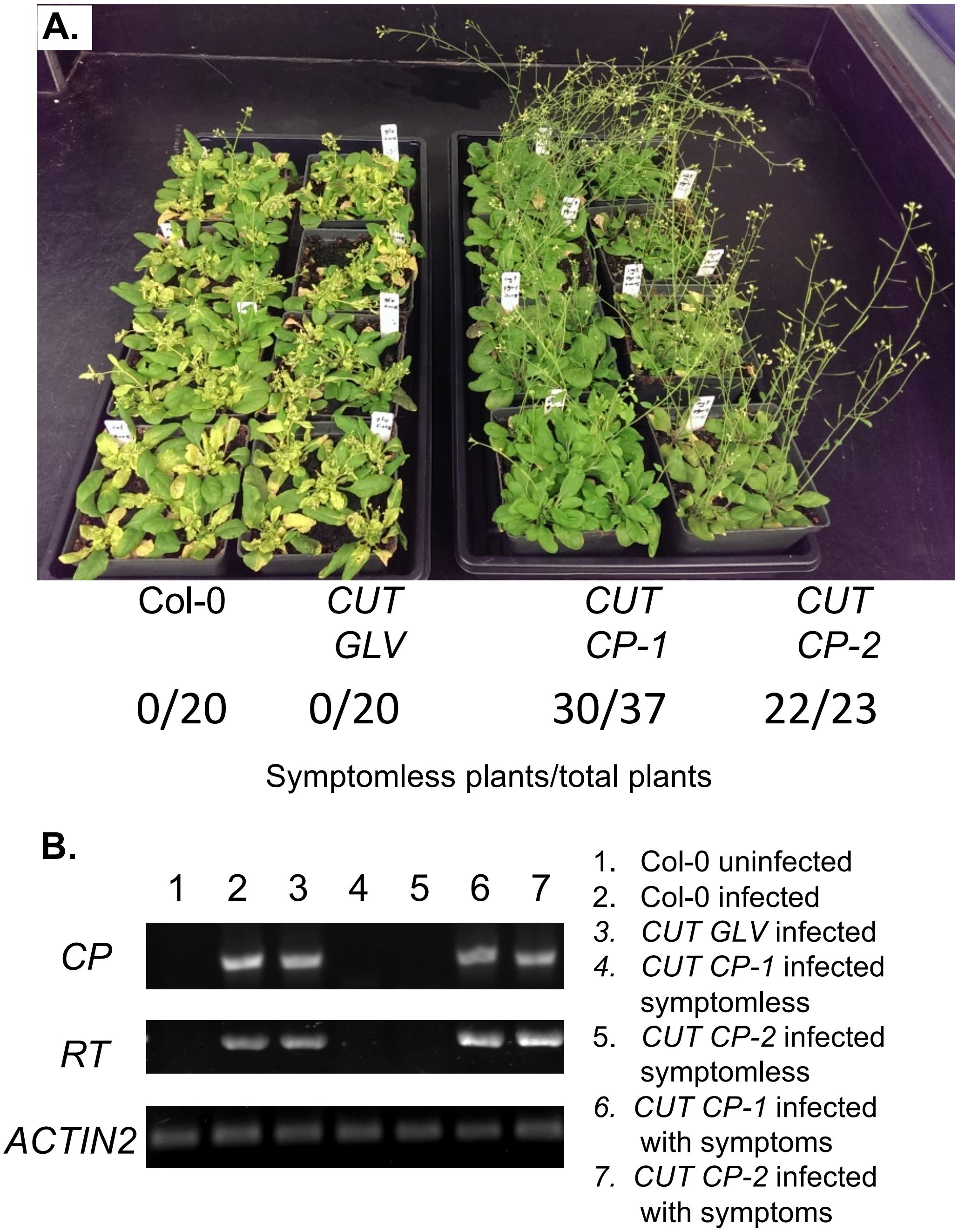

Figure S4. Cas9-mediated viral immunity by targeting the ORF of CaMV *CP*. A. CaMV-infected plants at 26 DPI. Both Col-0 (left) and *CUT GLV* (middle) transgenic plants display extensive symptoms of chlorosis and stunting, while most *CUT CP-1* or *CUT CP-2* transgenic plants remain symptomless. Numbers below summarize the number of plants without symptoms/total plants infected. B. The CaMV *CP* gene is detectable in Col-0, *CUT GLV* plants and *CUT CP* plants displaying symptoms, but not in symptomless *CUT CP* plants. *RT*, viral reverse transcriptase. Arabidopsis *ACTIN2* was used as a loading control.
