# Supplementary material for: CRISPR/Cas9‐mediated resistance to cauliflower mosaic virus
Source: Plant Direct. 2018 Mar 7;2(3):e00047. doi: 10.1002/pld3.47 (PMC6508564; doi:10.1002/pld3.47)
Supplement: Supplementary file 2 [file PLD3-2-e00047-s002.pdf]

Supplemental Table 1. Cas9-induced mutations in CaMV *CP* sequences from systemically infected tissue

Site: All nucleotide (nt) numbers are relative to wt CaMV *CP* sequence. Insertion and deletion are between the given nt numbers.

gRNA: G, gRNA. Numbering as in Supplemental Table 1.

Origin: Origin of inserted gene sequence. *CP* (w260).

Effect on CP protein: wt CP protein: 487 amino acids (aa).

Clone	Mutation in *CP* gene	Site (nt)	gRNA	Affected sequence	Origin	Effect on CP protein
**1A**	Single base deletion	330	G5	A		Frame shift after E110, stop codon 35 aa after, total length 145 aa
**2A**	142 bp deletion	257,400	G1, G2	TTCAGGAGAAGAACCTGAATTCGAGCAAGTTCGAATGGACCGAACAGGAG<br>GAACGGAGATTCCCAAAGAAGAAGATGGTGAAGGACCATCAAGATACAA<br>TGAGAGAAAGAGAAAGACCCCGGAGGACCGGTACTTTCCAACT		Frame shift after D86, stop codon 12 aa after, total length 98 aa
**10B**	6 bp deletion	326,333	G5	AGAAGA		In frame deletion of 2 aa (E109,E110)
**7B**	Single base insertion	330,331	G5	A		Frame shift after E110, stop codon 2 aa after, total length 112 aa
	106 bp insertion	330,331	G5	AGATGGTGAAGGACCATCAAGATACAATGAGAGAAAGAGAAAGACCCCG<br>GAGGACCGGTACTTTCCAACTCAACCAAAGACCATTCCAGGACAAAAGCA<br>GACGTCA	*CP*	
**9B**	Single base insertion	330,331	G5	A		Frame shift after E110, stop codon 2 aa after, total length 112 aa
