# Supplementary material for: CRISPR/Cas9‐mediated resistance to cauliflower mosaic virus
Source: Plant Direct. 2018 Mar 7;2(3):e00047. doi: 10.1002/pld3.47 (PMC6508564; doi:10.1002/pld3.47)
Supplement: Supplementary file 3 [file PLD3-2-e00047-s003.pdf]

January 25, 2018

Editors,
Plant Direct

Dear Editors:

I thank you and the reviewers for your insightful suggestions on our manuscript entitled: “CRISPR/Cas9-mediated resistance to cauliflower mosaic virus”. We are gratified that the reviewers considered our manuscript: “The data are substantial and further strengthening the concept that CRISPR-Cas9 can be used for durable resistance against DNA viruses in plants.” and “the study was well conducted, with the necessary controls and experiments performed with rigor and replicates. The manuscript was well-written, and the conclusions are justified by the results.”

We have revised the manuscript as suggested and provided sequences as requested by Reviewer 1. Below I summarize how we have responded to and incorporated the specific comments from the reviewers into the revised manuscript.

I have also included 2 text files, one with track of changes and the other one is a clean file as you have requested.

Thank you for your consideration and, if we can in any way assist your review, please contact me.

Sincerely,

Xiaofeng Wang

November 26, 2017

Xiaofeng Wang
Virginia Tech
213 Latham Hall
Blacksburg, Virginia 24061

RE: CRISPR/Cas9-mediated resistance to cauliflower mosaic virus

Dear Dr. Wang:

Thank you for submitting to Plant Direct. All required reviews have been returned and we have now finished our evaluation of your manuscript. In light of the reviewers' and editor's comments, further revisions are needed before the paper can be accepted for publication in Plant Direct.

Please address all revisions requested by Reviewer 1. The potential future experiment suggested by reviewer 2 would be of interest for you to consider in future studies.

Please view the editors' and reviewers' comments below and use their suggestions as a guide while you work on your revision.

When uploading the revised version of this article, please be sure to include the following:

-A word document that contains your response to the reviewers. You should respond to each reviewer comment and note the changes made to the manuscript. If you do not agree with a reviewer's comment and choose not to make a suggested revision, please explain why. Please try to provide as complete an answer as possible to each reviewer's criticisms.
-A tracked changes document with each change highlighted
- A clean version of the latest version of the manuscript

To upload your revision, please click the link below.
https://plantdirect.msubmit.net/cgi-bin/main.plex?el=A6Lr3Ca4A4GOs1I3A9ftdbdoGcu9GJQq7IzUx5y8sfgZ

In order to provide as timely a service as possible, we ask that your revision is resubmitted within three months after receipt of this request. If an extension is needed, please send a request, along with a brief explanation, to the editorial office at plantdirect@wiley.com .

Thank you very much for giving us an opportunity to review your work. I look forward to receiving the next version.

Sincerely,

Monica Höfte

Hsou-min Li

Editor, pd Site

-- Reviewer comments:
Reviewer #1 :

Liu et al, efficiently targeted CaMV, a pararetrovirus by a multiplex CRISPR/Cas9 system in planta. Targeting of coat protein of CaMV DNA in the plant nucleus rendered plants

symptomless of CaMV and this resistance was transgenerational in T2 and T3. Liu et al, also provide a comprehensive data about the small RNAs generated from the sgRNAs and confirmed that the observed interference is mainly Cas9-sgRNA dependent. Also, authors confirmed the escape of the mutated virus and their systemic movement. The data presented are interesting and up to the standard and would be interesting for plant direct readers.

I have some minor comments and suggestions to provide a clear picture to the scientific community if other researchers want to use this system for developing resistance to DNA viruses in plants.
1. Did the author observe modification at multiple sites in the same polynucleotide sequence? at least this information will confirm that a single genome can be targeted at multiple site.

We thank Reviewer for excellent inquiry. We did not identify extra editing events in the *CP* sequence in Fig. 3B and supplemental Table 1, other than what we have reported.

2. Throughout the manuscript gRNA need to be replaced with single guide RNA (sgRNA).

We thank Reviewer for the correction and we have changed gRNA to sgRNA.

3. Full map with complete sequence of CaMV and the vectors used must be provided as supplementary data.

We have provided maps (Figure S2 and 3) and sequences (pCUT CP.txt and pCUT CP No Cas.txt) of constructs *pCUT CP* and *pCUT CP No Cas*. We also provided full-length sequence of CaMV strain W260 as a text file: CaMV W260 full-length sequence.txt.

4. Did the author observe a differential CaMV interference among different lines or among T2 and T3 generations? If any, it must be discussed in the manuscript.

We used 2 transgenic lines and tested in T1, T2, and T3. We did not observe obvious differences between the two lines and among generations in resistance to CaMV infections.

The data are substantial and further strengthening the concept that CRISPR-Cas9 can be used for durable resistance against DNA viruses in plants.

Reviewer #2 :

Liu et al., 2017, Plant Direct
• This study demonstrates using the CSRISPR-Cas9 system to confer immunity to Cauliflower mosaic virus (CaMV), a double-stranded DNA pararetrovirus. The CRISPR-Cas9 mediated immunity has been demonstrated in the past for few other plant DNA viruses such as geminiviruses, however not for DNA pararetroviruses. So, this is the first report of CRISPR use for antiviral immunity against this important class of plant viruses.
• Overall, the study was well conducted, with the necessary controls and experiments performed

with rigor and replicates. The manuscript was well-written, and the conclusions are justified by the results.

• In addition to demonstrating the effectiveness of the CRISPR technology for antiviral immunity, the authors also clarified the interference of plant small RNA (siRNA) and RNAi pathways in the CRISPR efficiency in plants. The authors find no evidence to support that host RNAi could have caused the antiviral resistance, through the production of gRNA derived small RNA.

• Interestingly, the authors do show that there is production of 21-nt siRNAs from the 3' gRNA antisense strand (possibly produced by DCL4), thus raising the possibility that host RNAi could influence the gRNA accumulation and as such CRISPR efficiency. In this context, a potential future experiment I would suggest is to test if gRNA populations are affected in Arabidopsis loss-of-function mutants of DCL4 or RDRs.

We thank Reviewer 2 for insightful suggestion for future directions.
